# A controlled comparison of the BacT/ALERT® 3D and VIRTUO™ microbial detection systems

**DOI:** 10.1007/s10096-017-2994-8

**Published:** 2017-05-12

**Authors:** H. Totty, M. Ullery, J. Spontak, J. Viray, M. Adamik, B. Katzin, W. M. Dunne, P. Deol

**Affiliations:** 1 R&D Microbiology, bioMérieux, Inc., Durham, NC USA; 2 Biomathematics and Knowledge Engineering, bioMérieux, Inc., Hazelwood, MO USA; 3 Scientific Office, bioMérieux, Inc., Durham, NC USA

## Abstract

The performance of the next-generation BacT/ALERT® VIRTUO™ Microbial Detection System (VIRTUO™, bioMérieux Inc., Hazelwood, MO) was compared to the BacT/ALERT® 3D Microbial Detection System (3D, bioMérieux Inc., Durham, NC) using BacT/ALERT® FA Plus (FA Plus), BacT/ALERT® PF Plus (PF Plus), BacT/ALERT® FN Plus (FN Plus), BacT/ALERT® Standard Aerobic (SA), and BacT/ALERT® Standard Anaerobic (SN) blood culture bottles (bioMérieux Inc., Durham, NC). A seeded limit of detection (LoD) study was performed for each bottle type in both systems. The LoD studies demonstrated that both systems were capable of detecting organisms at nearly identical levels [<10 colony-forming units (CFU) per bottle], with no significant difference. Following LoD determination, a seeded study was performed to compare the time to detection (TTD) between the systems using a panel of clinically relevant microorganisms inoculated at or near the LoD with 0, 4, or 10 mL of healthy human blood. VIRTUO™ exhibited a faster TTD by an average of 3.5 h, as well as demonstrated a significantly improved detection rate of 99.9% compared to 98.8% with 3D (*p*-value <0.05).

**Electronic supplementary material:**

The online version of this article (doi:10.1007/s10096-017-2994-8) contains supplementary material, which is available to authorized users.

## Introduction

Each year in the USA, there are approximately 200,000 documented bloodstream infections [1]. Delayed laboratory diagnosis of bacteremia or fungemia can lead to increased mortality [2]. Therefore, rapid detection of microorganisms in blood and sterile body fluids is essential for optimized patient care. The BacT/ALERT® VIRTUO™ Microbial Detection System is the next generation of BacT/ALERT® instrumentation with an improved user interface capable of providing automated processes that were previously performed manually. The VIRTUO™ system consists of an incubator, agitation mechanism, robotic apparatus for automated loading and unloading of bottles, and a tactile graphical user interface. The versatile and scalable design of VIRTUO™ allows for enhanced bottle capacity with a smaller footprint in the laboratory. Employing the same colorimetric technology used in previous generations of BacT/ALERT® instruments, VIRTUO™ measures the change in pH of the media associated with microbial growth and CO_2_ production via a colorimetric sensor in each bottle and optically monitors the reflectance of the sensor over time [3]. The system stores and interprets these readings using algorithms embedded in the firmware and/or software. More importantly, VIRTUO™ uses a new algorithm designed to optimize test sensitivity and specificity, while significantly reducing the time to detection (TTD) of microbial growth when paired with BacT/ALERT® FA Plus, PF Plus, FN Plus, Standard Aerobic (SA), or Standard Anaerobic (SN) bottles.

The BacT/ALERT® bottles are each formulated to recover and detect a subset of clinically relevant microorganisms. SA culture bottles are intended for aerobic microorganisms, while the SN bottles contain media and atmospheric conditions designed for anaerobic microorganisms [4, 5]. FA Plus and PF Plus culture bottles contain antimicrobial neutralizing resins as well as media and atmospheric conditions suitable for aerobic and facultative microorganisms [6, 7]. PF Plus bottles recover and detect microorganisms when only a small volume of blood is available [16]. FN Plus culture bottles also employ antimicrobial neutralizing resins, while using media and atmosphere essential for anaerobes [8].

A series of seeded studies directly compared the instrumentation and growth-based detection algorithms of the 3D and VIRTUO™ systems. The limit of detection (LoD) was evaluated using a test set of clinically relevant organisms seeded into bottles to determine the lowest inoculum [colony-forming units (CFU) per bottle] in which a 95% detection rate is obtained. Once the LoD was established, a controlled seeded study was performed at/near the LoD for each test organism with different blood volumes to compare the detection rates and TTD between systems.

## Materials and methods

### Limit of detection

For each organism–inocula evaluated, 60 bottles were seeded. A single inoculum was prepared for each microorganism and tested in FA Plus, PF Plus, FN Plus, SA, and SN bottles (Table 1). BioBalls™ or colonies from solid media were serially diluted to achieve a target suspension of 10 CFU per mL. Bottles were inoculated with 0.5 mL of the target suspension. Plate counts were conducted for each inoculum before and after bottle inoculation. Thirty bottles were loaded into 3D and 30 were loaded into VIRTUO™. Once bottles flagged positive, they were promptly subcultured to confirm purity. Negative bottles remained in the instrument for 5 days until declared negative, at which time they were subcultured to verify the absence of microorganism.

**Table 1:** Limit of detection (LoD) test panel by bottle type

Bottle type	Microorganism	Strain
FA Plus and PF Plus	*Candida albicans*	ATCC 14053
	*Enterococcus faecalis*	NCTC 12697
	*Escherichia coli*	NCTC 12923
	*Haemophilus influenzae* ^a^	ATCC 10211
	*Pseudomonas aeruginosa*	ATCC 9027
	*Staphylococcus aureus*	NCTC 10788
	*Streptococcus pneumoniae*	ATCC 6305
FN Plus	*Bacteroides fragilis* ^c^	ATCC 25285
	*Clostridium perfringens*	NCTC 8789
	*Enterococcus faecalis*	NCTC 12697
	*Escherichia coli*	NCTC 12923
	*Staphylococcus aureus*	NCTC 10788
	*Streptococcus pneumoniae*	ATCC 6305
SA	*Aspergillus brasiliensis*	NCPF 2275
	*Candida albicans*	ATCC 14053
	*Enterococcus faecalis*	ATCC 12697
	*Escherichia coli*	NCTC 12923
	*Haemophilus influenzae* ^a^	ATCC 10211
	*Pseudomonas aeruginosa*	ATCC 9027
	*Staphylococcus aureus*	NCTC 10788
	*Streptococcus pneumoniae* ^b^	ATCC 6305
SN	*Bacteroides fragilis*	ATCC 25285
	*Clostridium perfringens*	NCTC 8789
	*Enterococcus faecalis*	NCTC 12697
	*Escherichia coli*	NCTC 12923
	*Staphylococcus aureus*	NCTC 10788
	*Streptococcus pneumoniae* ^b^	ATCC 6305

^a^Supplemented with 1 mL human blood

^b^Supplemented with 1 mL human blood for SA and SN only

^c^Supplemented with 4 mL human blood for FN Plus only

The LoD was defined for each organism/bottle/system as the lowest inocula that resulted in at least 95% of the bottles being declared positive. Based on a Poisson distribution, in a target inoculum of 3 CFU, approximately 5% of the bottles would not receive a single organism, 42% would receive less than 3 CFU, and 53% would receive 3 CFU or greater.

### Comparison of detection systems

Human blood was collected from healthy donors in tubes containing 0.35% sodium polyanethol sulfonate (SPS) in 0.85% sodium chloride and pooled prior to testing [9]. Prior to inoculation, bottles were supplemented at three blood volumes: 0 mL, representing sterile body fluid; 4 mL, representing a pediatric blood sample volume; and 10 mL, representing an adult blood sample volume (Table 2). Uninoculated bottles containing 0, 4, or 10 mL blood served as negative controls. Both systems received nine seeded bottles at three blood volumes, for a total of 27 replicates per microorganism and bottle type.

**Table 2:** Instrument and algorithm comparison panel by bottle type

Bottle type	Organism	Bottles per organism in 3D	Bottles per organism in VIRTUO™	Blood volume (mL)
FA Plus, PF Plus, and SA	*Abiotrophia defectiva*	27	27	0^a^, 4^b^, 10^c^
	*Aggregatibacter actinomycetemcomitans* ^d^			
	*Aspergillus fumigatus*			
	*Aspergillus brasiliensis*			
	*Campylobacter jejuni* ^e^			
	*Candida albicans*			
	*Candida glabrata*			
	*Candida krusei*			
	*Cardiobacterium hominis*			
	*Corynebacterium jeikeium* ^d^			
	*Cryptococcus neoformans*			
	*Eikenella corrodens* ^d^			
	*Enterobacter aerogenes*			
	*Enterococcus faecalis*			
	*Escherichia coli*			
	*Haemophilus influenzae* ^d^			
	*Klebsiella pneumoniae*			
	*Listeria monocytogenes*			
	*Micrococcus luteus*			
	*Neisseria meningitidis* ^d^			
	*Proteus vulgaris*			
	*Pseudomonas aeruginosa*			
	*Salmonella enterica*			
	*Serratia marcescens*			
	*Staphylococcus aureus*			
	*Staphylococcus epidermidis*			
	*Stenotrophomonas maltophilia*			
	*Streptococcus agalactiae*			
	*Streptococcus mitis*			
	*Streptococcus pneumoniae*			
	*Streptococcus pyogenes*			
FN Plus and SN	*Bacteroides fragilis*			
	*Bacteroides thetaiotaomicron*			
	*Bacteroides vulgatus*			
	*Clostridium perfringens*			
	*Clostridium septicum*			
	*Eggerthella lenta*			
	*Enterococcus faecalis*			
	*Escherichia coli*			
	*Fusobacterium nucleatum*			
	*Parvimonas micra*			
	*Peptoniphilus asaccharolyticus*			
	*Staphylococcus aureus*			
	*Streptococcus pneumoniae*			
	*Streptococcus pyogenes*			

^a^Reflects sterile body fluid sample

^b^Reflects pediatric patient blood volume

^c^Reflects adult patient blood volume

^d^Fastidious organisms not expected to grow in the absence of blood

^e^Microaerophilic organism not expected to grow in SA bottle type

Thirty-two aerobic microorganism and 14 anaerobic microorganisms were used to evaluate the performance of the detection systems. Each microorganism was serially diluted to a target inoculum of ≤ 30 CFU/bottle. Plate counts were performed on each inocula before and after each testing event to verify purity and appropriate microorganism density. Bottles were incubated until flagged positive or declared negative after 5 days. All inoculated negative bottles were examined for the presence of viable organisms by subculture. Two positive bottles from each test combination were randomly selected for subculture to verify purity of the inoculated bottles.

All possible differences between 3D and VIRTUO™ TTD were calculated for bottle type, organism, and fill volume using pairwise analysis. Only matched test bottles (bottle type, organism, and blood volume) for which data were available from both culture systems were included in the final comparison analysis. TTD detections were compared between each system at the level of bottle type, organism, and blood volume. Ratios were calculated from TTD results for each microorganism by dividing the TTD from VIRTUO™ by the TTD from 3D. A ratio less than 1 indicates an improvement in TTD on VIRTUO™. A ratio approximately equal to 1 indicates that the TTD on 3D and VIRTUO™ were equivalent. Detection rates between instrument types were compared using Fisher’s exact test.

## Results

### Limit of detection

A total of 479 seeded BacT/ALERT® bottles were tested in the 3D system. Of these, 473 (98.8%) were declared positive and confirmed by subculture. A total of 1616 seeded culture bottles were tested in the VIRTUO™ system, with 1605 (99.3%) declared positive. All negative bottles were determined to be true-negative results by terminal subculture. Both systems were capable of detecting less than CFU/bottle for all bottle types. There was no difference in the LoD of the VIRTUO™ system for different bottle types when compared to the established LoD of the 3D system (Supplemental Tables 1a–d).

### Comparison of time to detection between systems

The VIRTUO™ algorithm provided an overall mean reduction in TTD of approximately 3.48 h (Table 3). FN Plus bottles exhibited the greatest TTD improvement, with an average decrease of 3.90 h. The SA bottle type had the least TTD improvement, with an average decrease of 3.14 h.

**Table 3:** Time to detection (TTD) and detection rates from BacT/ALERT® 3D and VIRTUO™ by bottle type and blood volume

Bottle type	Blood volume (mL)	3D		VIRTUO™		TTD difference (3D - VIRTUO™)	
		# (+)/#	Detection rate (%)	# (+)/#	Detection rate (%)	Mean TTD difference (h)	95% CI for mean TTD difference (h)
FA Plus	0	227/239	95.0	233/233	100.0*	3.62	(3.37, 3.86)
	4^**^	271/272	99.6	275/275	100.0	3.94	(3.47, 4.41)
	10	273/278	98.2	279/279	100.0*	3.24	(2.96, 3.51)
	Combined	771/789	97.7	787/787	100.0*	3.59	(3.39, 3.80)
FN Plus	0	93/93	100.0	91/91	100.0	3.62	(3.14, 4.10)
	4	117/121	96.7	122/122	100.0^*^	3.78	(3.27, 4.29)
	10	110/115	95.7	114/114	100.0*	4.28	(3.93, 4.64)
	Combined	320/329	97.3	327/327	100.0*	3.90	(3.64, 4.17)
SA	0	215/215	100.0	213/213	100.0	2.35	(2.13, 2.56)
	4	276/276	100.0	279/279	100.0	3.47	(3.33, 3.61)
	10	270/270	100.0	276/276	100.0	3.38	(3.14, 3.62)
	Combined	761/761	100.0	768/768	100.0	3.14	(3.02, 3.25)
SN	0	125/125	100.0	123/123	100.0	4.65	(4.46, 4.84)
	4	124/124	100.0	116/117	99.1	4.02	(3.76, 4.29)
	10	119/120	99.2	119/120	99.2	2.20	(1.58, 2.82)
	Combined	368/369	99.7	358/360	99.4	3.65	(3.42, 3.88)
Overall		2220/2248	98.8	2240/2242	99.9*	3.48	(3.38, 3.58)

*Significant difference between detection rates; Fisher’s exact test *p*-value <0.05; ^**^represents PF Plus

Based on the comparison ratios (Table 4), all organisms had faster or equivalent TTD with VIRTUO™, except for *Stenotrophomonas maltophilia*, which generated slower TTD in FA Plus bottles (data not shown). Over half of the Gram-negative organisms tested had an average TTD of less than 24 h in the VIRTUO™ system, with an overall TTD ratio of 0.85. Overall, Gram-positive organisms demonstrated the greatest TTD improvement. *Abiotrophia defectiva* and *Corynebacterium jeikeium* exhibited the largest TTD reductions in VIRTUO™, with comparison ratios of 0.66 and 0.65, respectively (Table 4). Large decreases in TTD across both anaerobic bottle types were also observed in VIRTUO™ with *Clostridium perfringens* and *Clostridium septicum* (Table 4). Yeasts demonstrated smaller TTD improvements with VIRTUO™. Although the test panel for moulds was limited, the TTD of both *Aspergillus* species benefited from the VIRTUO™ detection algorithm (Table 4).

**Table 4:** Overall TTD comparisons between BacT/ALERT® 3D and VIRTUO™ by organism

Group	Organism	3D		VIRTUO™		Comparison ratio (VIRTUO™ TTD/3D TTD)
		Mean TTD (h)	Recovery rate (%)	Mean TTD (h)	Recovery rate (%)	
Gram-positive	*Abiotrophia defectiva*	25.7	100.0	17.0	100.0	0.66
	*Clostridium perfringens*	12.1	100.0	9.1	100.0	0.76
	*Clostridium septicum*	16.6	100.0	12.3	100.0	0.74
	*Corynebacterium jeikeium*	50.5	100.0	32.8	100.0	0.65
	*Eggerthella lenta*	35.3	94.4	34.7	96.2	0.98
	*Enterococcus faecalis*	14.2	100.0	11.3	100.0	0.80
	*Listeria monocytogenes*	21.7	100.0	20.2	100.0	0.93
	*Micrococcus luteus*	35.1	100.0	32.5	100.0	0.93
	*Parvimonas micra*	47.0	100.0	41.0	100.0	0.87
	*Peptoniphilus asaccharolyticus*	51.3	100.0	46.9	100.0	0.91
	*Staphylococcus aureus*	16.1	100.0	13.1	100.0	0.81
	*Staphylococcus epidermidis*	19.8	100.0	17.1	100.0	0.86
	*Streptococcus agalactiae*	15.1	100.0	11.6	100.0	0.77
	*Streptococcus mitis*	13.2	100.0	10.2	100.0	0.77
	*Streptococcus pneumoniae*	17.1	100.0	14.5	100.0	0.85
	*Streptococcus pyogenes*	15.3	100.0	12.6	100.0	0.82
Gram-negative	*Aggregatibacter actinomycetemcomitans*	46.5	100.0	43.4	100.0	0.93
	*Bacteroides fragilis*	34.7	100.0	32.4	100.0	0.94
	*Bacteroides thetaiotaomicron*	42.9	100.0	36.7	100.0	0.86
	*Bacteroides vulgatus*	43.3	100.0	40.7	100.0	0.94
	*Campylobacter jejuni*	46.1	100.0	41.6	100.0	0.90
	*Cardiobacterium hominis*	54.9	80.0	47.9	100.0	0.87
	*Eikenella corrodens*	26.2	100.0	22.5	100.0	0.86
	*Enterobacter aerogenes*	12.9	100.0	10.7	100.0	0.83
	*Escherichia coli*	11.4	100.0	9.0	100.0	0.79
	*Fusobacterium nucleatum*	53.9	84.4	43.7	100.0	0.81
	*Haemophilus influenzae*	20.0	100.0	16.9	100.0	0.85
	*Klebsiella pneumoniae*	12.2	100.0	9.7	100.0	0.80
	*Neisseria meningitidis*	22.3	100.0	19.3	100.0	0.86
	*Proteus vulgaris*	13.8	100.0	11.4	100.0	0.82
	*Pseudomonas aeruginosa*	17.6	100.0	14.3	100.0	0.81
	*Salmonella enterica*	13.6	100.0	11.1	100.0	0.82
	*Serratia marcescens*	15.1	100.0	11.3	100.0	0.75
	*Stenotrophomonas maltophilia*	29.4	100.0	36.6	100.0	1.25
Mould	*Aspergillus fumigatus*	34.9	100.0	30.2	100.0	0.87
	*Aspergillus brasiliensis*	58.4	83.0	46.3	100.0	0.79
Yeast	*Candida albicans*	27.3	100.0	26.1	100.0	0.95
	*Candida glabrata*	51.9	100.0	44.3	100.0	0.85
	*Candida krusei*	21.9	100.0	19.8	100.0	0.90
	*Cryptococcus neoformans*	63.2	100.0	63.2	100.0	1.00

### Comparison of detection rates between systems

In some instances, bottles inoculated at or near the LoD remained negative, likely due to the non-uniform distribution (Poisson distribution) of the inoculum. Only matched test bottles (bottle type, organism, and blood volume) for which data were available from both detection systems were included in the final comparison analysis.

A total of 2242 inoculated culture bottles were tested in the VIRTUO™ system, of which 2240 (99.9%) were declared positive. A total of 2248 inoculated bottles tested in the 3D system were available for comparison to VIRTUO™, of which 2220 (98.8%) were declared positive (Table 3).

For individual bottle types, only the rates of detection observed for SN bottles containing blood were not significantly different between systems (*p* > 0.05; Table 3). All other bottle types tested demonstrated a detection rate in the VIRTUO™ greater than or equal to the detection rate of 3D. All seeded SA, FA Plus, and FN Plus bottles were declared positive in the VIRTUO™ system (Table 4). Furthermore, FN Plus bottles in the 3D system exhibited decreased detection rates as blood volume increased, resulting in seven false-negative results for *Fusobacterium nucleatum* (Table 3). False-negative results were also observed in FA Plus bottles seeded with *Cardiobacterium hominis* and *Aspergillus brasiliensis* in the 3D system.

## Discussion

While testing at lower inoculum levels may delay TTD, comparing the detection systems at or near the LoD provides a more stringent evaluation compared to clinical samples. The data presented showed no difference in the LoD between 3D and VIRTUO™ for the panel of microorganisms tested. Even at low inoculum levels, 55% of the organisms evaluated in this study had an average TTD of less than 24 h in VIRTUO™. Further, our data demonstrates that the VIRTUO™ system provided a significantly improved rate of detection and faster TTD compared to the 3D (*p* < 0.05).

Overall, VIRTUO™ exhibited an improved detection rate of 99.9% compared to the 98.8% detection rate of 3D, with an average decrease in TTD of 3.48 h. FN Plus bottles had the most noticeable TTD improvement, with an average reduction of 3.90 h. Another seeded study reported significantly faster TTD with VIRTUO™ compared to 3D for several bacterial species in FA Plus and FN Plus bottles, with a median reduction of 2.8 h [10]. Additionally, the TTD for *Candida glabrata* in FA Plus bottles was reduced from a median of 65 h on 3D to 54 h on VIRTUO™ [10]. Previous studies demonstrate faster TTD for antibiotic-free samples and coagulase-negative staphylococci with BacT/ALERT® FA Plus and FN Plus bottles compared to BD BACTEC™ culture bottles [11–13]. The improved detection algorithm of the VIRTUO™ system could further enhance this advantage in cases where blood cultures are collected from patients receiving concurrent antimicrobial therapy [6, 8, 10].

Gram-positive organisms had the greatest reduction in TTD with VIRTUO™. In addition, VIRTUO™ improved the TTD for *Fusobacterium nucleatum* by an average of 1.8 days (31 to 57 h, depending on bottle type). Earlier case studies report that the mean number of days required to recover this organism from blood culture in the BACTEC™ system (BACTEC 9120, BD Diagnostic Systems) was 2.6 days (range 35–87 h) [12]. Additionally, the VIRTUO™ algorithm detected 100% of *F. nucleatum* in FN Plus bottles, as well as 100% of *Aspergillus brasiliensis* and *Cardiobacterium hominis* in FA Plus bottles, whereas for these same combinations, the 3D system generated a few false-negative results. Although our panel evaluated a limited subset of yeast and moulds, a high number of *Aspergillus* false-negatives have been observed using various blood culture systems, including the BACTEC™ and 3D systems [14]. In some instances, organisms may be encountered that grow in the culture bottle but do not produce sufficient CO_2_ to signal positive [4–8]. A previous report indicated that *C. hominis* generally produces small incremental changes to the growth indices in automated blood culture systems after 3–5 days and recommended incubation for at least 14 days before cultures are presumed negative [15, 16]. The improved algorithm of VIRTUO™ is capable of detecting these minute changes, reducing false-negative results, and allowing for an overall reduction in *C. hominis* TTD from 54.9 h in 3D to 47.9 h.

Automated blood culture systems provide improved detection of bacteremia and fungemia and, as a result, have become a staple in the diagnostic clinical microbiology laboratory. Our studies compared the instrumentation and growth-based algorithms of the 3D and VIRTUO™ systems using a panel of compatible blood culture bottles seeded with tightly controlled inoculum levels at/near the LoD and at multiple blood volumes. The VIRTUO™ algorithm improved positive blood culture TTD while reducing false-negative results when directly compared to the current 3D system. Further comparison studies performed with patient samples in a clinical setting are needed to validate the improved performance of VIRTUO™.

## Electronic supplementary material

ESM 1 (DOC 63 kb)
