# Supplementary material for: A controlled comparison of the BacT/ALERT® 3D and VIRTUO™ microbial detection systems
Source: Eur J Clin Microbiol Infect Dis. 2017 May 12;36(10):1795–800. doi: 10.1007/s10096-017-2994-8 (PMC5602088; doi:10.1007/s10096-017-2994-8)
Supplement: Supplementary file 1 — (DOC 63 kb) [file 10096_2017_2994_MOESM1_ESM.doc]

**Supplemental Table 1A:** FA Plus LoD for Test Organisms

| **Organism** | **BTA® 3D** | | | | **BTA® VIRTUO™** | | | |
| --- | --- | --- | --- | --- | --- | --- | --- | --- |
|  | **Inoculum CFU/bottle** | **Inoculum Range (CFU)** | **Percent Instrument Positive** | **Percent Subculture Positive** | **Inoculum CFU/bottle** | **Inoculum Range (CFU)** | **Percent Instrument Positive** | **Percent Subculture Positive** |
| *C. albicans* | 6 | 0 - 11 | 100 | 100 | 7 | 1 - 11 | 100 | 100 |
| *E. faecalis* | 4 | 1 - 7 | 100 | 100 | 4 | 1 - 9 | 100 | 100 |
| *E. coli* | 6 | 3 - 11 | 100 | 100 | 4 | 1 - 8 | 100 | 100 |
| *H. influenzae* | 8 | 4 - 12 | 100 | 100 | 3 | 2 - 4 | 100 | 100 |
| *P. aeruginosa* | 5 | 2 - 7 | 96.7 | 96.7 | 8 | 4 - 10 | 96.7 | 96.7 |
| *S. aureus* | 3 | 1 - 7 | 100 | 100 | 3 | 1 - 4 | 100 | 100 |
| *S. pneumoniae* | 4 | 1 - 8 | 100 | 100 | 3 | 2 - 6 | 98.3 | 98.3 |
| Negative control | N/A | | | | N/A | | | |

* Inoculum ranges are based on twelve colony count plates per test event

Supplemental Table 1B: FN Plus LoD for Test Organisms

| **Organism** | **BTA® 3D** | | | | **BTA® VIRTUO™** | | | |
| --- | --- | --- | --- | --- | --- | --- | --- | --- |
|  | **Inoculum CFU/bottle** | **Inoculum Range (CFU)** | **Percent Instrument Positive** | **Percent Subculture Positive** | **Inoculum CFU/bottle** | **Inoculum Range (CFU)** | **Percent Instrument Positive** | **Percent Subculture Positive** |
| *B. fragilis* | 5 | 2 - 7 | 100 | 100 | 4 | 2 - 5 | 96.7 | 96.7 |
| *C. perfringens* | 3 | 1 - 6 | 96.7 | 96.7 | 4 | 2 - 6 | 100 | 100 |
| *E. faecalis* | 4 | 2 - 7 | 100 | 100 | 3 | 1 - 5 | 100 | 100 |
| *E. coli* | 4 | 1 - 6 | 100 | 100 | 4 | 2 - 6 | 100 | 100 |
| *S. aureus* | 4 | 1 - 9 | 100 | 100 | 6 | 3 - 11 | 98.3 | 98.3 |
| *S. pneumoniae* | 6 | 3 - 8 | 100 | 100 | 5 | 3 - 7 | 100 | 100 |
| Negative control | N/A | | | | N/A | | | |

* Inoculum ranges are based on twelve colony count plates per test event

**Supplemental Table 1C: SA LoD for Test Organisms**

| **Organism** | **BTA® 3D** | | | | **BTA® VIRTUO™** | | | |
| --- | --- | --- | --- | --- | --- | --- | --- | --- |
|  | **Inoculum CFU/Bottle** | **Inoculum Range (CFU)** | **Percent Instrument Positive** | **Percent Subculture Positive** | **Inoculum CFU/Bottle** | **Inoculum Range (CFU)** | **Percent Instrument Positive** | **Percent Subculture Positive** |
| *A. brasiliensis* | 4 | 1 - 7 | 96.7 | 96.7 | 4 | 1 – 7 | 100 | 100 |
| *C. albicans* | 7 | 1 - 11 | 100 | 100 | 7 | 1 – 11 | 100 | 100 |
| *E. faecalis* | 4 | 1 - 9 | 100 | 100 | 4 | 1 – 9 | 100 | 100 |
| *E. coli* | 4 | 1 - 8 | 100 | 100 | 4 | 1 – 8 | 100 | 100 |
| *H. influenzae* | 3 | 2 - 4 | 100 | 100 | 3 | 2 – 4 | 98.3 | 98.3 |
| *P. aeruginosa* | 8 | 4 - 10 | 100 | 100 | 8 | 4 – 10 | 100 | 100 |
| *S. aureus* | 3 | 1 - 4 | 100 | 100 | 3 | 1 – 4 | 98.3 | 98.3 |
| *S. pneumoniae* | 3 | 2 – 6 | 96.6 | 96.6 | 3 | 2 – 6 | 96.7 | 96.7 |
| Negative control | N/A | | | | N/A | | | |

* Inoculum ranges are based on twelve colony count plates per test event

Supplemental Table 1D: SN LoD for Test Organisms

| **Organism** | **BTA® 3D** | | | | **BTA® VIRTUO™** | | | |
| --- | --- | --- | --- | --- | --- | --- | --- | --- |
|  | **Inoculum CFU/Bottle** | **Inoculum Range (CFU)** | **Percent Instrument Positive** | **Percent Subculture Positive** | **Inoculum CFU/Bottle** | **Inoculum Range (CFU)** | **Percent Instrument Positive** | **Percent Subculture Positive** |
| *B. fragilis* | 4 | 2 - 5 | 100 | 100 | 4 | 2 - 5 | 100 | 100 |
| *C. perfringens* | 4 | 3 - 6 | 98.3 | 98.3 | 4 | 2 - 6 | 100 | 100 |
| *E. faecalis* | 3 | 1 - 5 | 96.7 | 96.7 | 3 | 1 - 6 | 100 | 100 |
| *E. coli* | 4 | 2 - 6 | 100 | 100 | 4 | 2 - 6 | 98.3 | 98.0 |
| *S. aureus* | 6 | 3 - 11 | 100 | 100 | 6 | 3 - 11 | 100 | 100 |
| *S. pneumoniae* | 5 | 3 - 7 | 100 | 100 | 5 | 3 - 7 | 100 | 100 |
| Negative control | N/A | | | | N/A | | | |

* Inoculum ranges are based on twelve colony count plates per test event
